# Impact of rapid on-site evaluation on diagnostic accuracy of EUS-guided fine-needle aspiration of solid pancreatic lesions: experience from a single center

**DOI:** 10.1186/s12876-022-02330-w

**Published:** 2022-05-27

**Authors:** Irem Guvendir, Itir Ebru Zemheri, Kamil Ozdil

**Affiliations:** 1 Pathology Department, Umraniye Training and Research Hospital, University of Health Sciences, Alemdağ Bulvarı, Elmalıkent Mahallesi, 34766 Ümraniye, İstanbul, Turkey; 2 Gastroenterology Department, Umraniye Training and Research Hospital, University of Health Sciences, Alemdağ Bulvarı, Elmalıkent Mahallesi, 34766 Ümraniye, İstanbul, Turkey

**Keywords:** On-site evaluation, Pancreas, Cytopathology, EUS-FNA

## Abstract

**Background:**

The use of ROSE in EUS-FNA pancreatic lesions is still controversial in many centers. In this study, we aimed to demonstrate the contribution of ROSE to the diagnostic accuracy, as well as its assistance to the pathologist/cytopathologist.

**Methods:**

162 EUS-FNA biopsies were included in the study. EUS-FNA cytology results were reported according to the six-tiered system of Papanicolaou Cytopathology Society and compared to their final diagnosis with histopathology and/or clinical follow-ups regarding malignancy. The diagnostic yield, the difference in diagnostic accuracy, and the contribution of ROSE to providing the pathologist with adequate tissue uptake (number of slides and cell blocks) for further examination were compared in the ROSE and non-ROSE patient groups.

**Results:**

In the non-ROSE group, the diagnostic accuracy according to the final diagnoses was 96% and the sensitivity was 94.44%, specificity 100%, PPV 100%, NPV 87.50%; while diagnostic accuracy was 97.09%, sensitivity 97.47%, specificity 95.83%, PPV 98.77%, NPV 92% in patients with ROSE. There was no significant difference in diagnostic accuracy between those with and without ROSE (*p*: 0.078). In diagnostic cases, the number of passes, slides and cell blocks were significantly higher in patients with ROSE than those without ROSE (*p*: 0.003, *p*: 0.007, *p*: 0.012, respectively). ROSE was independently associated with diagnostic yield when evaluated by number of passes, slides, cell blocks in regression analysis (*p*: 0.001, OR: 5.07, confidence interval: 1.89–13.5).

**Conclusion:**

ROSE may increase the acquisition of sufficient tissue, but it does not have an advantage in diagnostic accuracy. ROSE may raise the number of slides, which may assist the pathologist for the diagnosis. If the lesion is solid and/or contains a solid component, diagnostic yield is higher in patients where ROSE is available. Therefore, ROSE still maintains its applicability in terms of increasing the diagnostic efficiency and making the final diagnosis.

## Background

Although pancreatic cancer is the 14th most common cancer in the world, it ranks 7th among cancer related deaths [1]. The reported 5-year survival rate for pancreatic cancer patients is quite low, about 6%, ranging from 2 to 9% [2]. Therefore, prompt diagnosis and early initiation of treatment of pancreatic cancers is very critical for patients.

Today, endoscopic ultrasound guided fine needle aspiration (EUS-FNA) biopsy greatly contributes to the early diagnosis of pancreatic cancers. The correct interpretation of the cytological material and the communication between the pathologist and the clinician are very important, and the Papanicolaou Society of Cytopathology in Pancreatobiliary System (PSC-PS), revised in 2014, makes significant contributions to this goal [3]. However, tissue acquisition and diagnostic accuracy under EUS guidance are affected by various factors and their influence on the diagnostic efficacy cannot be ignored. Type of needle, number of needle passes, endoscopist's experience, and rapid on-site evaluation (ROSE) are some of these factors. In addition, the higher amount of tissue obtained, better quality of the cytological material, and the availability of the cell block are among other factors that assist cytopathologists/pathologists [4, 5].

Regarding EUS-FNA, it can be argued that rapid on-site evaluation (ROSE) can greatly contribute to obtaining adequate tissue, and diagnostic efficacy and accuracy. As a matter of fact, when ROSE is not available diagnostic accuracy may be reduced by 10–15% [6]. In a meta-analysis, studies where ROSE was available had better sensitivity and slightly higher specificity compared to studies without ROSE [7]. However, many high-volume centers do not practice ROSE, since this process requires extra time and workforce but, low-volume centers still may need / practice it [8]. It is important to demonstrate the contribution of ROSE to clinical practice and to optimize the evaluation. The ideal number of passes, and especially the number of slides and cell blocks required for adequate tissue acquisition and high diagnostic accuracy when ROSE is available, has not been adequately studied in the literature.

Our aim in this study was (1) to evaluate the impact of ROSE’s availability on diagnostic accuracy of EUS-FNA biopsy of solid pancreatic lesions (2) to show the adequacy of tissue acquisition and diagnostic accuracy of EUS-FNA, if and how ROSE contributes to this (3) To investigate the effect of number of passes, number of slides and presence of cell block on diagnostic efficiency in EUS-FNA biopsies of pancreatic lesions when ROSE is available.

## Methods

Patients older than 18, who applied to the gastroenterology unit between 2016–2021 and underwent EUS-FNA biopsy for pancreatic lesion were evaluated retrospectively. The clinical follow-up of patients for at least 6 months after EUS-FNA biopsy, and the histopathological diagnoses obtained by surgery or tru-cut biopsy, if any, were analyzed. Patients whose follow-up were in other centers were evaluated using nation-wide medical electronic records. Patients’ oncology admissions, treatments (chemotherapy-radiotherapy regimens for pancreatic malignancy) and other pathology reports, radiological work-ups, surgical follow-ups were analyzed. Patients whose follow-up results could not be reached and who had purely cystic lesions were excluded from the study.

Patients’ histopathological diagnoses obtained by surgery or tru-cut biopsy were regarded as “final histopathology”. Patients who were diagnosed with pancreatic carcinoma clinically and / or histopathologically and received oncological treatment were regarded to have “malignant clinical diagnosis”.

A total of 162 EUS-FNA biopsies were included in the study. All EUS procedures were performed by the same experienced gastroenterologist. EUS-FNA cytology results were reported according to the six-tiered system of Papanicolaou Cytopathology Society (PSC-PC) [3]. Reported categories were as follows; “I- non-diagnostic”; “II- benign”; “III- atypical”; “IVa- neoplastic benign”; “IVb- neoplastic other”; “V- suspicion of malignancy”; “VI- malignant”. Cytological diagnoses (malignant / non-malignant) were compared with final histopathological and/or clinical diagnoses (malignant / non-malignant). “Diagnostic yield” was defined for cases which can be diagnosed other than category I according to PSC-PS. This study was approved by the ethics committee of the University of Health Sciences Umraniye Training and Research Hospital and meets the requirements of the Declaration of Helsinki.

### Technique

All patients provided informed consent for endoscopy (Fujinon Fujifilm, Tokyo, Japan, VP-4450 HD, EG 580 UT) and EUS-FNA. The EUS-FNA was performed using 22 G needle.

ROSE was provided by pathology residents, pathologists or cytopathology technicians present in the endoscopy unit. The patients were divided into two groups with and without ROSE; without ROSE patients was evaluated as “non-ROSE” group. One or more air-dried Diff-Quick stained smear was prepared from each pass and qualification were granted. Remaining tissues were spread on slides and fixed in ethanol. The thick, white-yellowish colored biopsy material remaining at the needle tip after smears was fixed in tubes filled with 10% formaldehyde. Then the slides, which were fixed in ethanol, were stained with Papanicolaou stain in the pathology laboratory. The biopsy material was processed and embedded in paraffin blocks, 4.5–5-micron sections were taken and stained with Hematoxylin–Eosin. Slides and cell block sections, were evaluated by two pathologists, immunohistochemical staining and molecular testing were performed when necessary.

### Statistical analysis

Sensitivity, specificity, positive predictive value (PPV), negative predictive value (NPV), and diagnostic accuracy of PSC-PS were calculated using cytological diagnosis (benign or malignant) with the final histopathological and/or clinical diagnosis (malignant or non-malignant). Non-diagnostic (category-I) cases were not included when calculating diagnostic accuracy. Based on distribution characteristics, results were expressed as mean ± standard deviation (SD) or median with interquartile range (IQR). The comparison of qualitative variables was analyzed using the Pearson Chi-square test or Fisher exact test; Quantitative independent variables were compared using t-test and Mann Whitney U test for parametric and non-parametric distributions respectively.

The results were evaluated in 95% confidence interval and statistical significance level was defined as *p* < 0.05. The analyzes were performed using IBM SPSS-21 (Statistical Package for Social Sciences, Chicago, IL, USA).

## Results

162 cases with known final diagnosis were evaluated. 102 (62%) of the patients were male and the mean age in the whole group was 60.54 (23–86). There was no age difference between women and men (59.7 ± 13.3, 61.8 ± 12.9 respectively, *p*: 0.6). In terms of EUS characteristics, 22 lesions were pure cystic, 36 lesions were cystic with accompanying solid components, and 104 lesions were pure solid.

ROSE was available for 121 cases, while it was not performed in 41 cases. The distribution of the lesions according to the anatomical regions of the pancreas were as follows; 33 in the uncinate, 71 in the head, 29 in the body, and 29 in the tail. There was no significant difference between the localizations of lesions according to the cystic / solid characteristic, the size of the lesions, or whether ROSE was performed.

The number of passes was significantly higher in patients with ROSE compared to patients without ROSE (median: 2, mean:1.79; 1, 1.37 respectively; *p*: 0.004). The median number of slides was also significantly higher in patients who underwent ROSE (Table 1). In addition, the median number of cell blocks obtained in patients with ROSE was 2, while it was 1 in patients without ROSE, and it was significantly higher in patients with ROSE (*p* < 0.001) (Table 1).

**Table 1:** Comparison of patients with/without ROSE by using passes of needles, slides and cell blocks

Number of	Patients with ROSE (n:121)	Patients without ROSE (non-Rose group)(n:41)	*p*
Passes of needles (mean-median min–max)	1.79 -2 1–5	1.37–1 1–6	0.004
Number of Slides (mean-median min–max)	9.9- 9 1–27	7.3–8 0–31	0.001
Number of Cell blocks (mean-median min–max)	2.1–2 0–6	0.8–1 0–5	< 0.001

Patients’ PSC-PC results were reported as; category-I for 34 patients, cat.-II for 30, cat.-III for 18, cat.- IVa for 3, cat.-IVb for 10, cat.-V for 11, and cat.-VI for 56 patients (Table 2). Thirty-four of the lesions were non-diagnostic (category I) and rest (n: 128) were considered as diagnostic (category: II, III, IVa, IVb, V, VI). The cases with ROSE showed higher "diagnostic yield" than those without ROSE, and ROSE was related to obtaining a result in a diagnostic PSC-PS category (*p*: 0.001).

**Table 2:** Distribution of the cases according to Papanicolaou Society of Cytopathology for Pancreatobiliary System (PSC-PS)

PSC-PS category	n:162	Biopsy or surgery procedure (n:26)	Concordance with definite histopathological or clinical diagnosis
I (non-diagnostic)	34(20%)	9	21 malignant, 13 benign
II (benign)	30(18.5%)	3	93.3% (3 of them false negative)
III (atypical)	18(11%)	4	94.4% (1 of them false positive)
IVa (neoplastic: benign)	3(0.18%)	0	%100
IVb (neoplastic: other)	10(0.61)	4	80% (3 of them had true positive but not concordant) *
V (suspicious for malignancy)	11(0.67%)	2	%100
VI (malignant)	56(34.5%)	4	%100

*One had high grade PANIN, one had ductal adenocarcinoma in their Whipple procedures. The other patient had accepted as pancreatic adenocarcinoma due to clinical follow ups

No significant difference was found between the diagnostic and non-diagnostic groups in terms of the number of passes, slides, and cell blocks (*p*: 0.79, 0.2, 0.09, respectively). In addition, in diagnostic cases ROSE was associated with higher number of passes, slides and cell blocks (*p*: 0.003, *p*: 0.007, *p*: 0.012, respectively). ROSE was also independently associated with diagnostic yield when evaluated together with number of passes, slides, cell blocks using regression analysis (*p*: 0.001, OR:5.07, confidence interval: 1.89–13.5).

Histopathological final diagnosis was obtained in 16% of the lesions (n:26), or determined according to the histopathological evaluation of tissue acquired from metastases, if any (Table 3). Accordingly, similar to various studies in the literature: categories II and IVa were regarded as benign (negative) while category III, IVb, V, VI were accepted as significant (positive) in terms of malignancy [9].

**Table 3:** Cases which have definitive histopathological diagnosis

No	Sex	Age	Location	S/C	Size (mm)	PSC- PS	Histopathology Specimen	Histopathology Diagnosis	Concordance
1	F	67	Head	C	28	I	Tru-cut	Panc. Adeno Ca	NA
2	F	62	Body	C	12	I	Tru-cut	Panc. Adeno Ca	NA
3	M	74	Tail	C	18	I	Sub.Pancreatectomy	Panc. Adeno Ca	NA
4	M	57	Tail	C	16	I	Tru-cut	Panc. Adeno Ca	NA
5	F	32	Tail	C	36	I	Whipple	Mucinous cystic neplasia	NA
6	K	61	Head	C	8	I	Whipple	Panc. Adeno Ca	NA
7	E	68	Body	C	59	I	Tru-cut	Panc. Adeno Ca	NA
8	F	33	Tail	C	48	I	Distal Pancreatectomy	Mucinous Cystic Neoplasia	NA
9	M	57	Tail	S	19	I	Whipple	Chronic Pancreatitis	T
10	M	65	Head	S	25	II	Whipple	Chronic Pancreatitis	T
11	M	57	Head	C	55	II	Tru-cut	Chronic Pancreatitis	T
12	F	54	Tail	S	30	II	Whipple	WON	T
13	M	55	Head	S	50	III	Tru-cut	Panc. Adeno Ca	T
14	M	73	Uncinate	C	35	III	Whipple	Panc. Adeno Ca	T
15	M	54	Head	S	64	III	Tru-cut	Follicular Lymphoma(G3b)	T
16	M	65	Body	S	16	III	Whipple	Panc. Adeno Ca	T
17	M	64	Uncinate	S	45	III	Whipple	Neuroendocrine Tumor(G3)	T
18	M	28	Head	S	20	III	Excision	Mixed Acinar Neuroendocrine Ductal Neoplasia	T
19	M	49	Uncinate	C	32	VI	Whipple	Panc. Adeno Ca	T
20	F	74	Head	S	28	VI	Whipple	Panc Adeno Ca	T
21	F	72	Head	C	32	VI	Whipple	Panc Adeno Ca	T
22	M	65	Tail	C	43	VI	Sub. Pancreatectomy	Ductal Adeno Ca	T
23	M	38	Head	S	27	IVb	Whipple	High Grade PANIN	F
24	M	37	Head	C	16	IVb	Whipple	IPMN	T
25	M	52	Tail	S	40	IVb	Whipple	Neuroendocrine Tumor(G1)	T
26	M	66	Head	S	22	IVb	Whipple	Ductal Adeno Ca	F

*S*: solid, *C*: cystic, *PSC-PS*: Papanicolaou Society of Cytology in Pancreatobiliary System, *NA*: not available, *T*: true, *F*: false, *WON*: Walled of pancreatic necrosis, *Sub*: Subtotal

Incompatibility with the final histopathological or clinical diagnosis was observed in 7 patients (0.54%). Three patients who were initially in category-II were diagnosed with pancreatic adenocarcinoma via histopathological examination of metastatic tissue, and one patient in category-III was regarded as chronic pancreatitis in their clinical follow-up. One of the two patients with category IVb was diagnosed with high grade PANIN on pathological examination of the Whipple resection, and the other with pancreatic ductal adenocarcinoma (Table-3). In addition, 2 patients in category IVb were diagnosed with pancreatic adenocarcinoma during clinical follow-up depending on clinical findings and presence of metastases.

When cytological diagnoses according to PSC-PS were compared with the final clinical and histopathological diagnoses in terms of malignancy, sensitivity was 96.9%, specificity was 96.8%, PPV was 98.9%, NPV was 90.9%, and diagnostic accuracy was 96.9%. For the group where ROSE was not available, the diagnostic accuracy was 96%, the sensitivity was 94.44%, specificity 100%, PPV 100%, NPV 87.50%. Regarding examinations with ROSE, diagnostic accuracy was 97.09%, sensitivity 97.47%, specificity 95.83%, PPV 98.77% and NPV was 92%. There was no significant difference in diagnostic accuracy between those with and without ROSE (*p*: 0.078).

## Discussion

One of our aims in this study was to assess the amount of tissue acquisition and diagnostic accuracy of EUS-FNA biopsy in pancreatic lesions. The compatibility of EUS-FNA PSC-PS categories with final clinical diagnosis or histopathology was quite high and parallel to the literature [10]. Discordancy of EUS-FNA cytology with final diagnoses was observed in 7 of 128 cases. ROSE was not available for 3 of these 7 patients. In a 57-year-old male patient, dense lymphocytes and histiocytes and a small number of ductal epithelial cells in cytology materials were evaluated in favor of chronic pancreatitis, reported as category-II and malignancy couldn’t been excluded; However, the final diagnosis was accepted as malignancy during the clinical follow-up and patient received chemotherapy. Another 49-year-old male patient with category-II PSC-PS, had degenerated epithelial cells in cytological examination. ROSE was performed for this patient, however because of high clinical suspicion of malignancy, a second EUS-FNA biopsy was performed. The second biopsy resulted as category-V and the patient was treated as adenocarcinoma. 67-year-old female was diagnosed as category-II instead of non-diagnostic category because of the observed, even if, small number of honeycomb epithelial cells. This patient with extensive metastases was diagnosed with pancreato-biliary adenocarcinoma as a result of tru-cut biopsy of metastatic liver lesion. A 54-year-old male patient had a history of chronic pancreatitis and walled of necrosis, was diagnosed as category-III. Cytology was interpreted as possible reactive findings due to the low number of atypical cells observed. Again, in the cytological materials of three patients who were diagnosed as category IVb and had clinical-cytological discordancy; diffuse mucin, anisochoric, increased nucleus size, and atypical epithelial cells were present. The final diagnoses of these patients during follow-up were pancreatic cancer, high-grade PANIN, and pancreatic adenocarcinoma, and all were diagnosed histo-pathologically with Whipple materials.

Studies have focused mainly on solid lesions of the pancreas and report conflicting results about the effect of ROSE on diagnostic accuracy [11]. To a lesser extent, ROSE for cystic lesions of the pancreas has not been shown to have any significant effect on diagnostic accuracy, and there are even some studies emphasizing that ROSE should not be performed on cystic lesions [12]. Some reported data in the literature point that the demographic characteristics of the patients and the location of the lesions in the pancreas do not affect ROSE [13]. Also; in this study, patient's age, size of the lesions, and distribution of the lesion did not differ with availability of ROSE.

Another purpose of our study was to evaluate the necessity of ROSE. Since, the role of ROSE for adequate tissue acquisition with EUS-FNA biopsy still remains as one of the most discussed issues in practice. In centers where it is available, ROSE can facilitate material acquisition in difficult situations and thus provide suitable specimen for histological evaluation that will combine the benefits of cytology and histology. Iglesias-Garcia et al. showed that ROSE was associated with significantly fewer passes, fewer scant samples, higher diagnostic yield, and higher diagnostic accuracy for diagnosing malignancy [13]. Furthermore, Klapman et al. compared the EUS-FNA cytology results obtained by the same endosonographer at two centers, with and without ROSE. The diagnostic yield (regarding malignancy) was higher when a cytopathologist was present (58% vs 41.5%; *p*: 0.006), with a lower number of inadequate tissue specimens (9% vs 20%; *p*: 0.035) [14]. However, on the contrary, Wani et al. compared the diagnostic yield and proportion of inadequate specimens undergoing EUS-FNA of pancreatic masses with (n: 121) and without (n: 120) ROSE, there was no difference between groups in the diagnostic yield (with ROSE 75.2% vs without ROSE 71.7%; *p*: 0.53) and proportion of inadequate specimens (9.9% vs 13.3%; *p*: 0.4). Procedures with ROSE had significantly lower number of passes (3.7 vs 7; *p* < 0.001). The overall procedure time, adverse events, number of repeat procedures, and cytologic characteristics of specimens were similar between groups [15]. Wani et al. in another review stated that, ROSE does not impact the diagnostic yield for malignancy and the number of inadequate specimens, based on the available evidence. Therefore, they recommended that ROSE should be used in centers that have difficulty in achieving tissue adequacy [16].

In this respect, the necessity of ROSE is still controversial. Some high-volume centers do not use ROSE due to its burden on time, human resources and workforce; but some studies emphasize the superiority of ROSE [3, 5]. The diagnostic accuracy of EUS-guided tissue acquisition under ROSE is reported to be higher than 90% in most studies; however, comparable results have also been reported from some trials without ROSE [13, 17]. In one recent meta-analysis, authors found that there was no indication that ROSE improved the diagnostic yield (risk difference [RD], 0.04; 95% CI, 0.05–0.13) [18]. In addition to these discussions, in this study, we showed that ROSE had a significant advantage over the non-ROSE group for diagnostic results, in the first EUS-FNA biopsies. However, although ROSE was found to be significant in terms of yielding diagnostic results, the final diagnostic accuracy of the cases with ROSE was not significantly different from those without ROSE.

Lisotti A. et al. showed in their study that, when EUS-FNA biopsy diagnoses and final diagnostic results were compared in cases with and without ROSE; performing with ROSE had a higher pooled sensitivity with similar pooled specificity. In particular, sensitivity and specificity were 83% (95% CI, 64–93%) and 98% (95% CI, 80–100%) when ROSE was present, respectively, as compared with 65% (95% CI), 57–73%) and 94% (95% CI, 31–100%) when ROSE was not available. [7]. However, Fabbri et al. pointed to no significant difference in sensitivity, specificity, positive and negative likelihood ratios, and diagnostic accuracy in his study, when 333 pancreatic solid lesions were divided into ROSE and non-ROSE. [11]. In addition, some studies reported that ROSE's success in providing diagnostic results is insufficient even if it is applied in non-diagnostic cases [17]. In our study, among 34 non-diagnostic cases, ROSE did not significantly change the success of acquiring final diagnostic results.

ROSE can also contribute to the pathologist in terms of the number of passes, slide smearing and cell blocks. In this study, the amount of tissue acquired with ROSE (number of slides, cell blocks) was found to be significantly superior than those without ROSE. The reason for this may be the intervention of the cytopathologist/ pathologist/ cytology technician who qualifies the cytological material as insufficient during ROSE, increasing the number of passes and the manipulation for obtaining tissue, also spreading the tissue to the slides and forming the appropriate cell block. The advantages of obtaining a cell block, especially with fine needle aspiration, are: being able to see the lesion pattern, performing immunohistochemical staining to support the diagnosis, and acquirement of tissue for molecular analysis, even contributing to the appropriate chemotherapy regimen and molecular targeted therapy [19].

Studies report that the needle type used and macroscopic on-site evaluation by the endoscopist effects the quality and tissue acquisition and thus success in acquiring cell block [20]. There is limited evidence on the diagnostic performance of EUS-guided fine-needle biopsy (FNB) sampling for GI subepithelial lesions and pancreas. Facciorusso A. et al. studied on a meta-analysis to compare EUS-FNB sampling performance with EUS-FNA [23]; For GI subepithelial lesions, pooled rate of obtaining adequate sample for FNB was 94.9% (range, 92.3–97.5%) and for FNA 80.6% (range, 71.4–89.7%; OR, 2.54; 95% CI, 1.29–5.01; P Z 0.007). When ROSE was available, no significant difference between the 2 techniques was observed. Optimal histologic core procurement rate was 89.7% (range, 84.5–94.9%) with FNB and 65% (range, 55.5–74.6%) with FNA (OR, 3.27; 95% CI, 2.03–5.27; *p* < 0.0001). In another study, S.F.Crino et al. suggested that EUS-FNB should become standard of care for grading assessment of suspected pNETs. Study showed stronger correlation for Ki-67 values between EUS-FNB and surgical specimens, and that EUS-FNB outperformed EUS-FNA in the evaluation of small pNETs [24]. In FNA, although white, yellowish-colored biopsy material of appropriate thickness helps to acquire more cells, and to show the pattern and architecture of the lesion, the role of slide smear for achieving a definitive diagnosis cannot be ignored [4, 13, 21]. Adequate slide number and presence of cell block may be sufficient to make a definitive diagnosis without the need for a second interventional procedure. In addition, it is very critical that the cell block can provide the needed extra tissue for auxiliary immunohistochemical techniques and molecular tests [22].

There are studies in the literature to optimize the needle type, number of passes, smearing slide techniques and cell block acquisition when ROSE is available [5, 6]. Erickson et al. in their study, stated that the number of passes should be between 3 and 6 in order to obtain high diagnostic accuracy with ROSE during the FNA procedure targetting pancreas [6]. Furthermore, Chung et al. in their multicenter study, suggested 4 as the optimum number of passes to be applied in centers that do not use ROSE in Korea [5]. Mizutani et al. demonstrated the contribution of the cell block acquirement to make the definitive diagnosis, in terms of the applicability of ancillary tests and molecular studies [19]. Therefore, even a single cell block from the lesion can be very valuable, increasing the sensitivity and specificity for accurate diagnosis. In addition, there are studies on the optimization of the slide smear technique; However, the optimum "number of slide smearing" requiring for high (a) diagnostic yield has not been examined in-depth for both ROSE and non-ROSE applications.

In our study, the number of passes, slides and cell blocks were significantly higher with ROSE application in diagnostic cases compared to those without ROSE. However, in cases with ROSE, the diagnostic cases was not different than non-diagnostic ones regarding the number of passes, slides and cell blocks. ROSE was independently associated with diagnostic yield in regression analysis, which can be interpreted as follows: (1) Although there is a pathologist at the bedside, it may not have sufficiently increased the number of passes and thus the tissue acquisition in non-diagnostic cases. In addition, (2) slides and cell blocks may have been prepared technically improperly, and it may have caused degeneration of cells and tissues with artefactual changes. This could have prevented giving diagnostic competence. (3) If the possibility of complications is high in patients who have undergone intervention, the number of passes may have been reduced.

## Conclusion

As a result, ROSE can increase the number of passes, slides and cell blocks, but it does not have an advantage in diagnostic accuracy. The optimum number of slides has not been examined in the literature, and ROSE may increase the number of required slide smears, which may benefit the pathologist in diagnosis. If the lesion is solid and/or contains a solid component, the success of obtaining a diagnostic yield is higher in patients with ROSE than in those without. Therefore, ROSE still maintains its applicability in terms of making the final diagnosis to the patient and increasing the diagnostic efficiency.

## Data Availability

The datasets (original extraction sheets) used and/or analysed during the current study are available from the corresponding author on reasonable request.

## Abbreviations

ROSE: Rapid on-site evaluation
EUS: Endosonographic ultrasound imaging
FNA: Fine needle aspiration
PSC-PS: Papanicolaou Society of Cytopathology in Pancreatobiliary System
PPV: Positive predictive value
NPV: Negative predictive value
SD: Standard deviation
IQR: Interquartile range
PANIN: High grade pancreatic intraepithelial neoplasia
IPMN: Intraductal papillary mucinous neoplasm
WON: Walled of necrosis
NA: Not available
T: True
F: False
